# Data resource profile: the National Health Insurance Research Database (NHIRD)

**DOI:** 10.4178/epih.e2018062

**Published:** 2018-12-27

**Authors:** Liang-yu Lin, Charlotte Warren-Gash, Liam Smeeth, Pau-Chung Chen

**Affiliations:** 1 Department of Non-communicable Disease Epidemiology, Faculty of Epidemiology and Population Health, London School of Hygiene and Tropical Medicine, London, UK; 2 Institute of Occupational Medicine and Industrial Hygiene, National Taiwan University College of Public Health, Taipei, Taiwan; 3 Department of Public Health, National Taiwan University College of Public Health, Taipei, Taiwan; 4 Innovation and Policy Centre for Population Health and Sustainable Environment, National Taiwan University College of Public Health, Taipei, Taiwan; 5 Department of Environmental and Occupational Medicine, National Taiwan University College of Medicine and Hospital, Taipei, Taiwan; 6 Office of Occupational Safety and Health, National Taiwan University Hospital, Taipei, Taiwan

**Keywords:** Database, Electronic health records, Information storage and retrieval, National Health Insurance Research Database, Taiwan

## Abstract

Electronic health records (EHRs) can provide researchers with extraordinary opportunities for population-based research. The National Health Insurance system of Taiwan was established in 1995 and covers more than 99.6% of the Taiwanese population; this system’s claims data are released as the National Health Insurance Research Database (NHIRD). All data from primary outpatient departments and inpatient hospital care settings after 2000 are included in this database. After a change and update in 2016, the NHIRD is maintained and regulated by the Data Science Centre of the Ministry of Health and Welfare of Taiwan. Datasets for approved research are released in three forms: sampling datasets comprising 2 million subjects, disease-specific databases, and full population datasets. These datasets are de-identified and contain basic demographic information, disease diagnoses, prescriptions, operations, and investigations. Data can be linked to government surveys or other research datasets. While only a small number of validation studies with small sample sizes have been undertaken, they have generally reported positive predictive values of over 70% for various diagnoses. Currently, patients cannot opt out of inclusion in the database, although this requirement is under review. In conclusion, the NHIRD is a large, powerful data source for biomedical research.

## INTRODUCTION

The increasing availability, size, and detail of electronic health records (EHRs) offer unprecedented opportunities for research. The advantages of EHRs include increased statistical power, speed, wide breadth, relatively low cost, representative population coverage, completeness of follow-up, and the ability to assess interventions in routine clinical care [1]. Linking EHRs to disease registries and other resources can further extend their utility. Meanwhile, randomised controlled trials (RCTs) control for known and unknown confounding factors; therefore, they are regarded as the gold standard for measuring the efficacy of interventions [2]. However, the true effectiveness of exposures may be influenced by many factors in a real-world setting, leading to a gap between efficacy and effectiveness. Consequently, real-world data, collected in non-RCT settings, are essential to bridging this gap [3]. As an important source of real-world data, EHRs have become a practical tool in medical research. By utilising EHRs, researchers can measure treatment effects, demonstrate trends in disease incidence and prevalence, and further explore possible disease aetiologies.

Among national EHR databases all over the world, the National Health Insurance Research Database (NHIRD) of Taiwan is unique. This large database, which contains data from 23 million residents of Taiwan, was previously described by Chen et al. [4]. However, the NHIRD was updated in 2016. This database changed its regulatory administration, was integrated with other datasets for further linkage, and released its full population dataset. The NHIRD now provides greater flexibility for scientific research. In this article, we introduce the latest version of the NHIRD, demonstrate its key features for research, and describe its strengths and weaknesses.

## BASIC DATA RESOURCES

### National Health Insurance programme of Taiwan

To increase the affordability and accessibility of health care, in 1995, the Taiwanese government initiated a single-payer health insurance system, known as National Health Insurance (NHI). NHI has a contract with most healthcare facilities in Taiwan, and it is mandatory for physicians to upload the claims data from each visit to the National Health Insurance Ministry. Notably, the primary care system in Taiwan is different from that of many other countries. Referrals from general practitioners are not required to receive specialist care; therefore, patients with non-emergency health concerns can either visit local private or public clinics or go directly to specialists at hospital outpatient departments [5]. In 2017, 93% of healthcare facilities in Taiwan contracted with NHI, except some self-pay private clinics [6]. As a programme that provides universal care health coverage, NHI covers all necessary medical expenses including outpatient visits, the inpatient system, prescriptions, treatment with traditional Chinese medicine, dental services, operations, and investigations such as X-rays or magnetic resonance imaging. The coverage of NHI reached 92% as it was established; by the end of 2014, NHI covered 99.9% of the Taiwanese population [7,8].

### History of insurance data usage and governance

**Figure 1.**
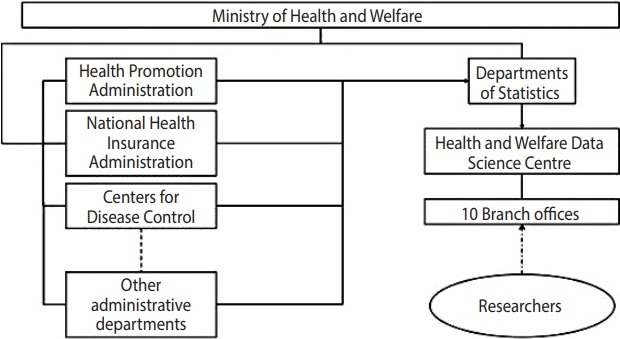
Administrative structure of the National Health Insurance Research Database [10].

In 2000, the anonymous and encrypted sampling dataset from this national insurance system was first released for use in research, under the regulation and maintenance of the National Health Research Institutes of Taiwan. From 2000 to 2013, the National Health Research Institutes made available to researchers general sampling datasets with 1 million subjects, as well as disease-specific sampling datasets. In 2016, these insurance data were moved to the Data Science Centre of the Ministry of Health and Welfare of Taiwan, where data are regulated and managed by the government [9]. The regulatory structure of the NHIRD is illustrated in Figure 1 [10]. The claims data from the NHI are stored and processed at the Data Science Centre, along with other governmental surveys and datasets. At the Data Science Centre, the NHIRD and other datasets are compared with the Household Registry Record from the Ministry of the Interior for quality control. Variables, such as sex and dates, are examined to ensure accuracy and consistency across different years. All data are de-identified and encrypted to protect participants’ privacy [11].

### Research using the National Health Insurance Research Database data

**Figure 2.**
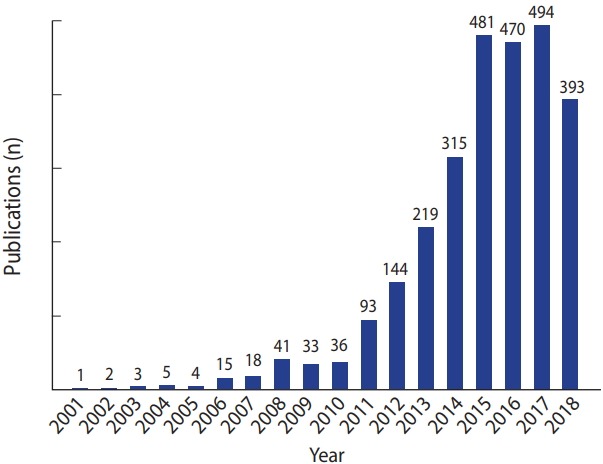
Publications using the National Health Insurance Research Database from 2000 to 2018.

The NHIRD is a powerful for observing chronic diseases and assessing the effects of treatments. For instance, hepatitis B virus (HBV) and hepatitis C virus (HCV) infections are relatively prevalent in Taiwan. Previous studies using the NHIRD demonstrated that the use of statins, medicines for decreasing low-density lipoprotein in the blood, was associated with a decreased incidence of hepatocellular carcinoma (HCC) in HBV and HCV patients [8,9]. Another study showed that the use of nucleoside analogues as antiviral treatments for chronic hepatitis B reduced HCC recurrence in HBV patients receiving liver resection [12]. Furthermore, the availability of data linkage makes it possible to conduct population-based studies of rare diseases. By using NHIRD data, Kuo et al. [13] demonstrated an increased heritable risk of systemic lupus erythematosus (SLE) and other autoimmune diseases among families of SLE patients. Since 2014, more than 300 published studies have used NHIRD data each year (Figure 2). To date, over 2,700 peer-reviewed studies have been published using NHIRD data, covering such topics as general medicine, multidisciplinary science, psychiatry, clinical neurology, oncology, and public environmental and occupational health.

## MEASUREMENTS

### Practice and patient data

**Figure 3.**
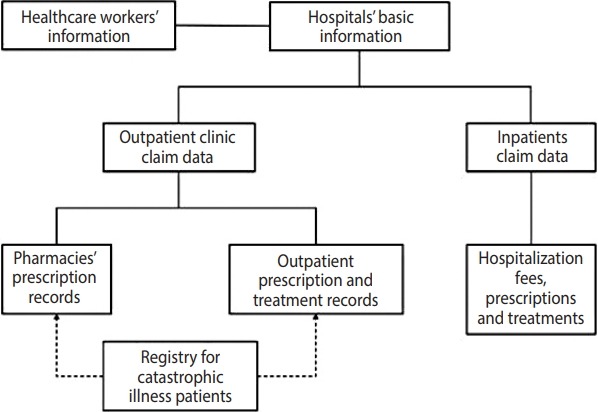
Data structure of the National Health Insurance Research Database.

The basic structure of the NHIRD data is shown in Figure 3. The de-identified data contain demographic variables, including the insured persons’ registration location, sex, age, investigations, diagnoses, prescriptions, and details of each outpatient visit or their inpatient care. Disease diagnoses are coded using the International Classification of Diseases, Ninth Revision. Each subject in the dataset is coded with an encrypted identifier, which can be used to link future patient data. Detailed laboratory test results and medical notes are not included in this database.

### Data release

The NHIRD data are released in three forms. The first form is a general dataset containing 2 million patients. Two million subjects are collected using stratified random sampling by age, sex, and the registry of regions from the full database population. They were sampled at three different time points: 2000, 2005, and 2010 (Supplementary Material 1). Each dataset contains claims data including diagnoses, prescriptions, investigation items, and treatments that the subjects received from 2000 to 2016. For the datasets sampled in 2005 and 2010, two additional datasets are available: from 2005 to 2016 and from 2010 to 2016. In addition to the complete claims data, these sampling datasets also contain data from cause of death datasets, cancer registry datasets, major illness datasets, and hospital information datasets. The general 2-million-patient sampling dataset is considered to be nationally representative.

The second form of NHIRD data are disease-specific databases. These databases contain complete claims data of all patients with a certain health condition. For instance, all patients with a diabetes diagnosis from 2002 to 2015 are included in the diabetes database. As of 2018, there are 13 disease-specific databases available for research (Table 1). These datasets can also be linked to cancer registry data and cause of death data.

The third form of NHIRD data is the full population dataset, which has been available for research since 2016. The full population dataset covers the entire Taiwanese population from 2000 to 2016, which comprises approximately 23 million people. Researchers can apply for complete claims data, including inpatient and outpatient records, investigations, and treatment, which can be linked with hospital information, birth certificate applications, death records, the cancer registry dataset, and the major illness datasets. Furthermore, the full population data can also be linked with individual datasets, a feature that will be introduced later. These released datasets are a valuable source for epidemiological research.

### Data linkage

Since 2016, under the authorisation and regulation of the Ministry of Health and Welfare, NHIRD data can be more widely linked with other public surveys at the Data Science Centre. These datasets include governmental surveys, disease registries, health surveys, social reporting system data, and welfare registry data. Detailed descriptions of these databases are given in Table 2. These databases and the NHIRD can be linked through an encrypted personal ID using deterministic record linkage. Due to privacy issues, this data linkage can only be processed by researchers at the Data Science Centre. Accessing some sensitive data, such as the domestic violence database, requires special authorisation from other administrative departments. In addition to linking governmental data, with the informed consent of study subjects, researchers are also allowed to link their own research databases with the NHIRD. For instance, the Taiwan Biobank Database is a national cohort containing biological samples and comprehensive examinations of 200,000 adult volunteers that will be linked to the NHIRD [14]. Such data linkages can help researchers discover possible interactions among genes, environmental factors, and diseases.

### Strengths and weaknesses

#### Strengths

The NHIRD is a nationally representative cohort that contains detailed registry and claims data from all 23 million residents of Taiwan. This huge database provides researchers with powerful and generalisable real-world evidence for biomedical studies. For instance, a molecular epidemiological study has suggested that aristolochic acid (AA), an ingredient in Chinese herbal remedies, was correlated to HCC in Taiwan and other Asian countries [15]. Similar findings were later found using NHIRD data. Chen et al. [16] analysed NHIRD data and discovered that using Chinese herbs containing AA increased the risk of HCC among patients with HBV infections. In addition, after the update in 2016, the NHIRD can be further linked with other datasets to increase the power and potential to research specific population subgroups, rare conditions, and factors that are not usually contained in clinical databases, such as living conditions, violence, or detailed lifestyle data. Payment and reimbursement data are also valuable for health economic analyses.

#### Weaknesses

There are some issues with the NHIRD. First, the NHIRD lacks comprehensive validation, although some validation studies of the clinical diagnoses in the NHIRD have been done. Some of these validation studies used national disease registries as the reference standard, which is more convincing. Other studies used hospital-based records to validate the diagnoses found in the NHIRD and have reported relatively high positive predictive values (over 70%) (Supplementary Material 2). However, the samples of these studies were small and drawn from a limited number of hospitals. Therefore, the samples may not be regarded as nationally representative. To improve the accuracy of the NHIRD, the Ministry of Health and Welfare of Taiwan recently initiated a national validation project using existing registry data [17]. However, until this new evidence of the database’s validity is reported, researchers should carefully interpret results from the NHIRD. Second, consent from the participants included in the NHIRD is another controversial issue. By law, all residents in Taiwan are required to have NHI, and their data are included in the NHIRD; there is currently no way for participants to opt out of this national cohort. However, in 2017, the Supreme Administrative Court upheld the legitimacy of using the NHIRD data for research [18]. People’s ability to opt out of inclusion in the NHIRD remains under discussion [19]. Finally, records of self-pay healthcare and out-of-pocket payments, such as for cosmetic surgery, are not included in the NHIRD. This may narrow the scope of research using the NHIRD, and researchers must be aware of the effects of these non-included variables.

### Data access

Researchers can access NHIRD data after ethical and scientific review processes. Prior to applying, researchers must obtain approval from the institutional review board. Notably, the applicant must be Taiwanese or be affiliated with a Taiwanese research institute. Applicants should submit their research proposal to the Data Science Centre. Proposals should include specific methods and variables required for their analyses. The cost of accessing data depends on the number of variables requested and the time period that they require the data for analyses; for example, accessing one variable for 1 year would cost 200 new Taiwanese dollars. After receiving an application, the Ministry of Health and Welfare reviews the legitimacy of the proposal, which is later reviewed by a scientific committee consisting of three experts. If one of the committee members disagrees with the proposed use of the data, then the researchers must submit a revised proposal to a higher advisory committee for a second review.

After receiving approval, researchers must go to the branches of the Data Science Centre to perform their data analyses. The analyses of NHIRD data are complicated, and there is no structural training course for using the NHIRD. Therefore, a mock dataset containing 100,000 subjects is provided by the Data Science Centre to help researchers in writing statistical analysis syntax. When researchers enter the Data Science Centre, they are allowed to use provided computers and software including SAS, Stata, R, and SPSS to conduct their data analyses [20].

### Ethics and confidentiality

Ethical review board approval is mandatory when applying to use NHIRD data. There are 27 institutional review boards capable of issuing approvals, and all are supervised and regulated by the Ministry of Health and Welfare [21]. To protect individuals’ confidentiality, all datasets in the Data Science Centre are pseudonymised. Personal ID, birth date, and names are encrypted, and this de-identification process was approved by an independent third party organisation [20]. To further secure the participants’ privacy, NHIRD datasets cannot be accessed outside the Data Science Centre, meaning that researchers must analyse these datasets at the Data Science Centre. When accessing the Data Science Centre, researchers are not allowed to bring any recording devices, including paper and pen. In addition, their statistical analysis syntax needs to be reviewed by the Data Science Centre prior to using the computers and software provided. The analysed results are also examined by the Data Science Centre before exporting. Any results with fewer than 3 subjects are not allowed to be exported to prevent re-identification [22].

## CONCLUSION

The NHIRD of Taiwan contains a large quantity of claims data and has the potential for multiple data linkages. Although more validation research is needed, and regulatory work to protect privacy is ongoing, this nationwide cohort is a valuable resource for medical research.

## Figures and Tables

**Table 1.** Disease-specific databases of the National Health Insurance Research Database

Database name	Year	No. of new case
Colorectal Cancer Health	2002-2015	175,405
Breast Cancer Health	2002-2015	136,476
Prostate Cancer Health	2002-2015	53,937
Systemic Lupus Erythematosus Health	2002-2015	29,637
Hypertension Health	2002-2015	3,342,827
Brain Tumour Health	2002-2015	10,267
Chronic Kidney Disease Health	2002-2015	1,066,892
End-Stage Renal Disease Health	2002-2015	134,228
Diabetes Mellitus Health	2002-2015	1,720,602
Injury	2000-2015	25,925,939
Triple-High^1^	2001-2015	6,558
Disability Process	1996, 1999, 2003, 2007, 2011	6,935
Maternal and Child Health	2004-2014	2,171,765

1 Triple-High: hypertension, hyperglycaemia, hyperlipidaemia.

**Table 2.** Databases available for linkage

Name of database	Year
Health data	
Taiwan Cancer Registry	2007-2012
Cause of death data	1971-2014
Birth certificate applications	2001-2013
Traffic accident data	2003-2014
“Triple-high Status” Survey	2006-2007
Taiwan Birth Cohort Study	2005
“Knowledge, Attitude, and Practice of Contraception” Survey	1965-2008
Taiwan Youth Health Survey File	2006-2010
Rare disease data	2012
Artificial reproductive data	1998-2012
Cancer screening – Pap smear data	2004-2013
Colorectal cancer screening	2010-2013
Breast cancer screening	2004-2013
Oral mucosal screening	2010-2013
Taiwan Healthy Behaviour Risk Factor Surveillance Survey File	2007-2012
Social surveys	
National Aboriginal Population Profile	2006-2012
Personal data for the sampled NHI claims cohorts	2000, 2005
National Health Interview Survey	2001-2009
Taiwan Longitudinal Study on Aging	1998-2011
Taiwan Smoking Behaviour Survey	2004-2009
Welfare databases	
The Juvenile Condition Survey in Taiwan-Fuchien Area	2003
Report of the Home Care Subsidy User Condition Survey	2007
The Satisfaction with Home Care Services Survey	2011
The Low-Income and Middle-Income Family Living Condition Survey	2013
Taiwan Longitudinal Study on Aging	2009-2013
Physically and Mentally Disabled Citizens Living and Demand Assessment Survey	2011
Single Parent Family Condition Survey	2010
Women’s Living Conditions Survey	1998-2011
Disabled Population Profile	2014
Low-income and middle-low-income household data	2014
Family violence data	2011-2014
Reported data of protection of children and youths	2011-2014
Reported data of sexual assault	2011-2014

NHI, National Health Insurance.

## SUPPLEMENTARY MATERIAL

Supplementary Material 1: Table S1 is available at http://www.e-epih.org/.

Supplementary Material 2: Table S2 is available at http://www.e-epih.org/.
