# Supplementary material for: Data resource profile: the National Health Insurance Research Database (NHIRD)
Source: Epidemiol Health. 2018 Dec 27;40:e2018062. doi: 10.4178/epih.e2018062 (PMC6367203; doi:10.4178/epih.e2018062)
Supplement: Supplementary file 1 [file epih-40-e2018062-supplementary1.pdf]

Supplementary Material 1

Table S1. Details of two million subjects datasets

Sampling Year	Total Number*	Sampling Number	Percentage	Following Period
2000	21,400,826	2,000,118	9.35%	2000-2016
2005	22,314,647	2,000,120	8.96%	2000-2016, 2005-2016
2010	23,074,487	2,000,126	8.67%	2000-2016, 2010-2016

*Data extracted from NHI annual statistics report in 2000, 2005, and 2010.
