# Supplementary material for: Data resource profile: the National Health Insurance Research Database (NHIRD)
Source: Epidemiol Health. 2018 Dec 27;40:e2018062. doi: 10.4178/epih.e2018062 (PMC6367203; doi:10.4178/epih.e2018062)
Supplement: Supplementary file 2 [file epih-40-e2018062-supplementary2.pdf]

Supplementary Material 2

Table S2. Validation Research about NHIRD

Author and Year	Disease	Methods	Results
Lin et al., 2005 (Lin et al., 2005)	Diabetes mellitus	Sampling patient-based survey	DOR:74.6%
Cheng et al,2011 (Cheng et al., 2011)	Ischemic stroke	Cross-sectional study comparing with one hospital records	PPV:88-94%
Chen et al., 2012 (Chen et al., 2012)	Epilepsy	Linking data from a community-based survey	Sensitivity:81.37% Specificity:99.83%
Shen et al, 2012 (Shen et al., 2012)	Pleural infection	Medical records of 50 randomly selected patients from a hospital	PPV: 88%
Shen et al, 2012 (Shen et al., 2012)	First-attack acute pancreatitis	Medical records of 50 randomly selected patients from a hospital	PPV: 88%
Cheng, 2014 (Cheng et al., 2014)	Acute myocardial infarction	Cross-sectional study comparing with one hospital records	PPV: 88%
Wu et al., 2014 (Wu et al., 2014)	Primary aldosteronism	Reviewing medical charts of all patients with the diagnosis of primary aldosteronism	Sensitivity: 89% Specificity: 88%

Chen et al., 2015 (Chen et al., 2015)	Tuberculosis	Cross matching data with a national survey and TB registry	Sensitivity: 86.9%<br>Specificity: 99.9 %
Cheng, 2015 (Cheng et al., 2015)	In-hospital mortality due to acute myocardial infarction and stroke	Comparing NHIRD coding with electronic medical record of one hospital and Catastrophic Illness Registry Data	PPV: 95%
Hsieh et al, 2015 (Hsieh et al., 2015)	Acute ischemic stroke	Comparing NHIRD with Taiwan Stroke Registry data	PPV: 88.4%
Lee et al., 2015 (Lee et al., 2015)	Aortic aneurysm and aortic dissection	Independent validation using medical record from a tertiary medical center	PPV: 92%
Yang et al., 2015 (Yang et al., 2015)	Allopurinol hypersensitivity	Review medical record from a medical center	33 cases linked to NHIRD were confirmed as allopurinol hypersensitivity
Liu et al., 2016 (Liu et al., 2016)	Parkinson's disease	Independent validation using medical record from a medical center	Sensitivity: 97.6%<br>Specificity: 92.3%<br>PPV: 98.8%<br>NPV: 85.7%
Sung et al, 2016 (Sung et al., 2016)	Hypertension (HTN), diabetes mellitus (DM), hyperlipidemia, atrial fibrillation (AF), coronary artery disease (CAD)	Matching stroke registry data from three hospitals with NHIRD	PPV:<br>HTN: 88.5%<br>DM: 92.0%<br>Hyperlipidemia: 89.5%<br>AF: 71.1%<br>CAD: 47.6%
Kao et al., 2017 (Kao et al., 2017)	Cancer diagnosis	Linking the National Cancer Registry database to NHIRD	PPV:<br>All cancer: 94%<br>10 Specific cancers: from 82% to 95 %

Lee et al., 2017 (Lee et al., 2017)	Psoriasis	Independent validation by reviewing medical charts of patient from a hospital	PPVS: 98.5%
Su et al., 2017 (Su et al., 2017)	Tuberculosis	Review medical record from a medical center	Sensitivity:96.3%
Chang et al., 2018 (Chang et al., 2018)	Varicose vein	Review medical records of randomly selected patients from three medical center	PPV: 98%

DOR: Diagnostic odds ratio; PPV: positive predictive value; NPV: negative predictive value

CHANG, S. L., HUANG, Y. L., LEE, M. C., HU, S., HSIAO, Y. C., CHANG, S. W., CHANG, C. J. & CHEN, P. C. 2018. Association of Varicose Veins With Incident Venous Thromboembolism and Peripheral Artery Disease. *Jama,* 319**,** 807-817.

CHEN, C. C., CHEN, L. S., YEN, M. F., CHEN, H. H. & LIOU, H. H. 2012. Geographic variation in the age- and gender-specific prevalence and incidence of epilepsy: analysis of Taiwanese National Health Insurance-based data. *Epilepsia,* 53**,** 283-90.

CHEN, C. C., CHIANG, C. Y., PAN, S. C., WANG, J. Y. & LIN, H. H. 2015. Health system delay among patients with tuberculosis in Taiwan: 2003-2010. *BMC Infect Dis,* 15**,** 491.

CHENG, C. L., CHIEN, H. C., LEE, C. H., LIN, S. J. & YANG, Y. H. 2015. Validity of in-hospital mortality data among patients with acute myocardial infarction or stroke in National Health Insurance Research Database in Taiwan. *Int J Cardiol,* 201**,** 96-101.

CHENG, C. L., KAO, Y. H., LIN, S. J., LEE, C. H. & LAI, M. L. 2011. Validation of the National Health Insurance Research Database with ischemic stroke cases in Taiwan. *Pharmacoepidemiol Drug Saf,* 20**,** 236-42.

CHENG, C. L., LEE, C. H., CHEN, P. S., LI, Y. H., LIN, S. J. & YANG, Y. H. 2014. Validation of acute myocardial infarction cases in the national health insurance research database in taiwan. *J Epidemiol,* 24**,** 500-7.

HSIEH, C. Y., CHEN, C. H., LI, C. Y. & LAI, M. L. 2015. Validating the diagnosis of acute ischemic stroke in a National Health Insurance claims database. *J Formos Med Assoc,* 114**,** 254-9.

KAO, W.-H., HONG, J.-H., SEE, L.-C., YU, H.-P., HSU, J.-T., CHOU, I. J., CHOU, W.-C., CHIOU, M.-J., WANG, C.-C. & KUO, C.-F. 2017. Validity of cancer diagnosis in the National Health Insurance database compared with the linked National Cancer Registry in Taiwan. *Pharmacoepidemiology and drug safety*.

LEE, C. C., LEE, M. T., CHEN, Y. S., LEE, S. H., CHEN, Y. S., CHEN, S. C. & CHANG, S. C. 2015. Risk of Aortic Dissection and Aortic Aneurysm in Patients Taking Oral Fluoroquinolone. *JAMA Intern Med,* 175**,** 1839-47.

LEE, M. S., YEH, Y. C., CHANG, Y. T. & LAI, M. S. 2017. All-Cause and Cause-Specific Mortality in Patients with Psoriasis in Taiwan: A Nationwide Population-Based Study. *J Invest Dermatol,* 137**,** 1468-1473.

LIN, C. C., LAI, M. S., SYU, C. Y., CHANG, S. C. & TSENG, F. Y. 2005. Accuracy of diabetes diagnosis in health insurance claims data in Taiwan. *J Formos Med Assoc,* 104**,** 157-63.

LIU, C. C., LI, C. Y., LEE, P. C. & SUN, Y. 2016. Variations in Incidence and Prevalence of Parkinson's Disease in Taiwan: A Population-Based Nationwide Study. *Parkinsons Dis,* 2016**,** 8756359.

SHEN, H. N., LU, C. L. & LI, C. Y. 2012. Epidemiology of pleural infections in Taiwan from 1997 through 2008. *Respirology,* 17**,** 1086-93.

SU, V. Y., SU, W. J., YEN, Y. F., PAN, S. W., CHUANG, P. H., FENG, J. Y., CHOU, K. T., YANG, K. Y., LEE, Y. C. & CHEN, T. J. 2017. Statin Use Is Associated With a Lower Risk of TB. *Chest,* 152**,** 598-606.

SUNG, S. F., HSIEH, C. Y., LIN, H. J., CHEN, Y. W., YANG, Y. H. & LI, C. Y. 2016. Validation of algorithms to identify stroke risk factors in patients with acute ischemic stroke, transient ischemic attack, or intracerebral hemorrhage in an administrative claims database. *Int J Cardiol,* 215**,** 277-82.

WU, V. C., HU, Y. H., WU, C. H., KAO, C. C., WANG, C. Y., YANG, W. S., LEE, H. H., CHANG, Y. S., LIN, Y. H., WANG, S. M., CHEN, L. & WU, K. D. 2014. Administrative data on diagnosis and mineralocorticoid receptor antagonist prescription identified patients with primary aldosteronism in Taiwan. *J Clin Epidemiol,* 67**,** 1139-49.

YANG, C. Y., CHEN, C. H., DENG, S. T., HUANG, C. S., LIN, Y. J., CHEN, Y. J., WU, C. Y., HUNG, S. I. & CHUNG, W. H. 2015. Allopurinol Use and Risk of Fatal Hypersensitivity Reactions: A Nationwide Population-Based Study in Taiwan. *JAMA Intern Med,* 175**,** 1550-7.
